# Supplementary material for: Model structures amplify uncertainty in predicted soil carbon responses to climate change
Source: Nat Commun. 2018 Jun 4;9:2171. doi: 10.1038/s41467-018-04526-9 (PMC5986763; doi:10.1038/s41467-018-04526-9)
Supplement: Supplementary file 1 — Supplementary Information [file 41467_2018_4526_MOESM1_ESM.pdf]

Model structures amplify uncertainty in predicted soil carbon responses to climate change

Shi et al.

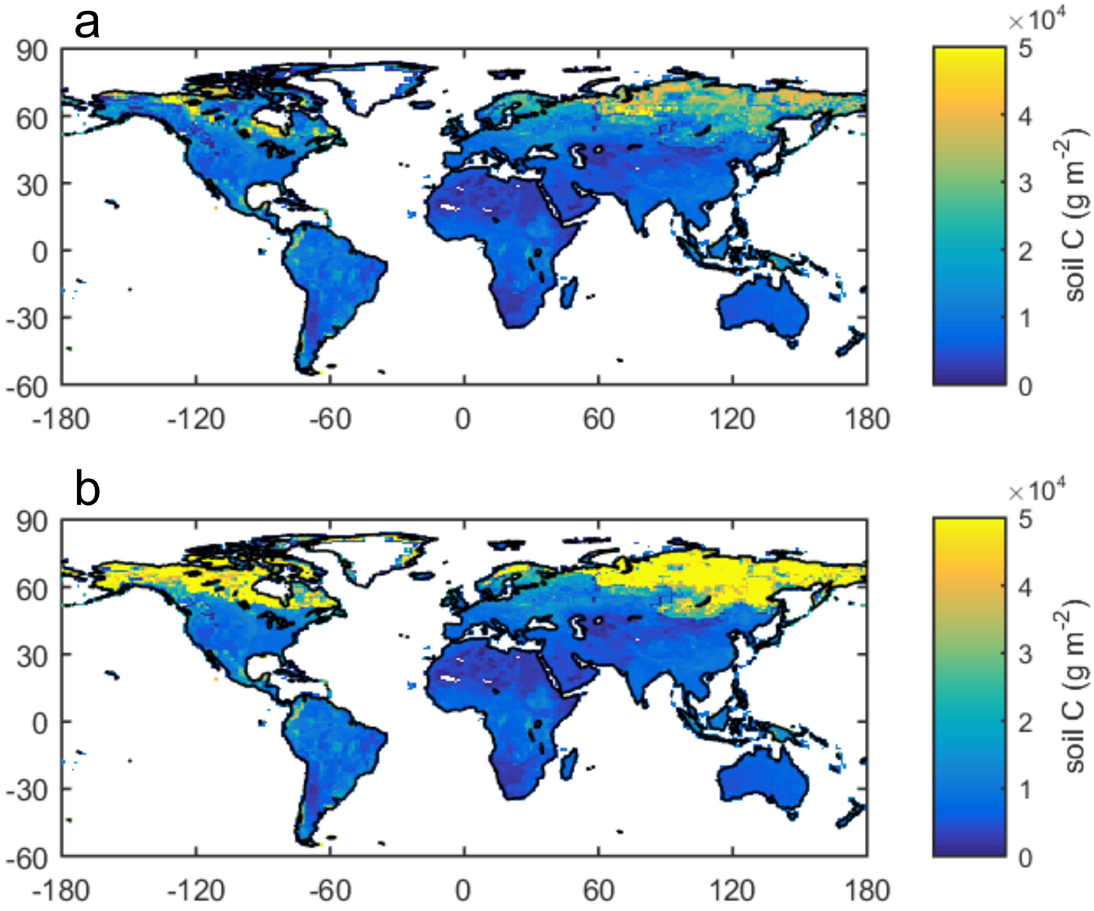

Supplementary Figure 1. Re-gridded soil carbon content from Harmonized World Soil Database (HWSD) and the Northern Circumpolar Soil Carbon Database (NCSCD) at the resolution of Community Land Model (1.25 × 0.94°). Note that the two figure panels are composite product based on both HWSD and NCSCD. Panel a shows the soil carbon content at 0 -100 cm depth, with NCSCD in the permafrost regions and HWSD in the rest of the land area; panel b shows the soil carbon content, with NCSCD in the permafrost regions down to 300 cm and HWSD in the rest of the land area down to 100 cm;

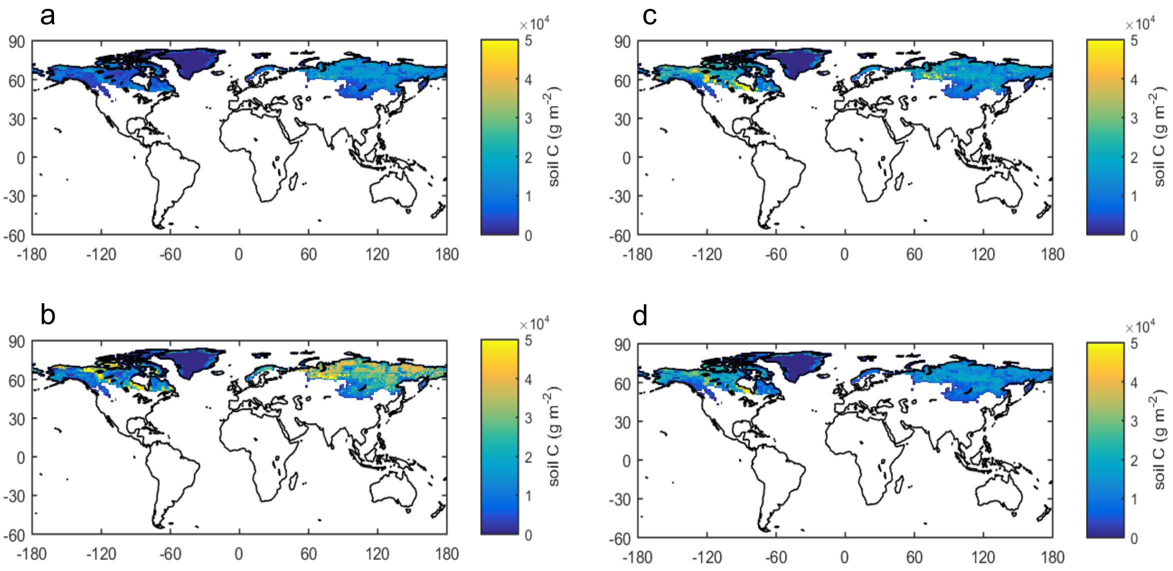

Supplementary Figure 2. The Northern Circumpolar Soil Carbon Database at the resolution of Community Land Model (1.25 × 0.94°) at different depths. a: 0-30 cm; b: 0-100 cm; c: 100-200 cm; d: 200-300 cm.

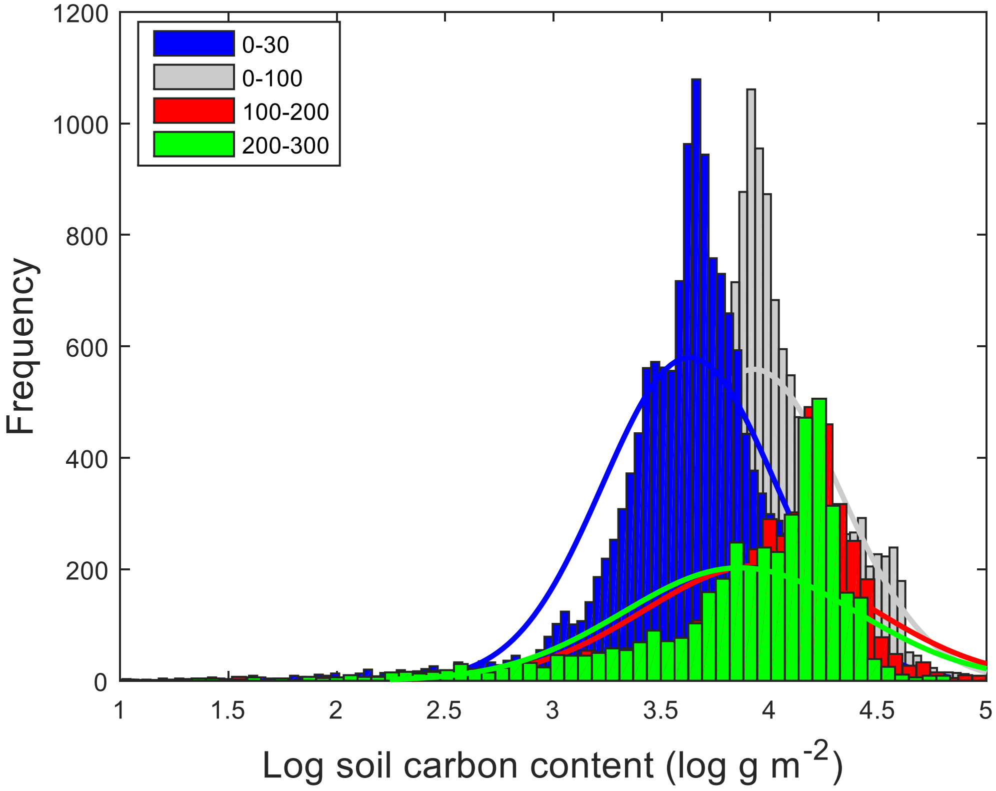

Supplementary Figure 3. Normal distribution of logged observational soil carbon content (composite database with HWSD and NCSCD) in the 0-30 cm, 0-100 cm, 100 – 200 cm, and 200 – 300 cm. The database is comprised by HWSD (down to 1 meter deep) in non-permafrost and NCSCD (down to 3 meters deep) in the permafrost. We used this composite database to conduct data assimilation. Data for 100-200 and 200-300 cm layers is only available in NCSCD.

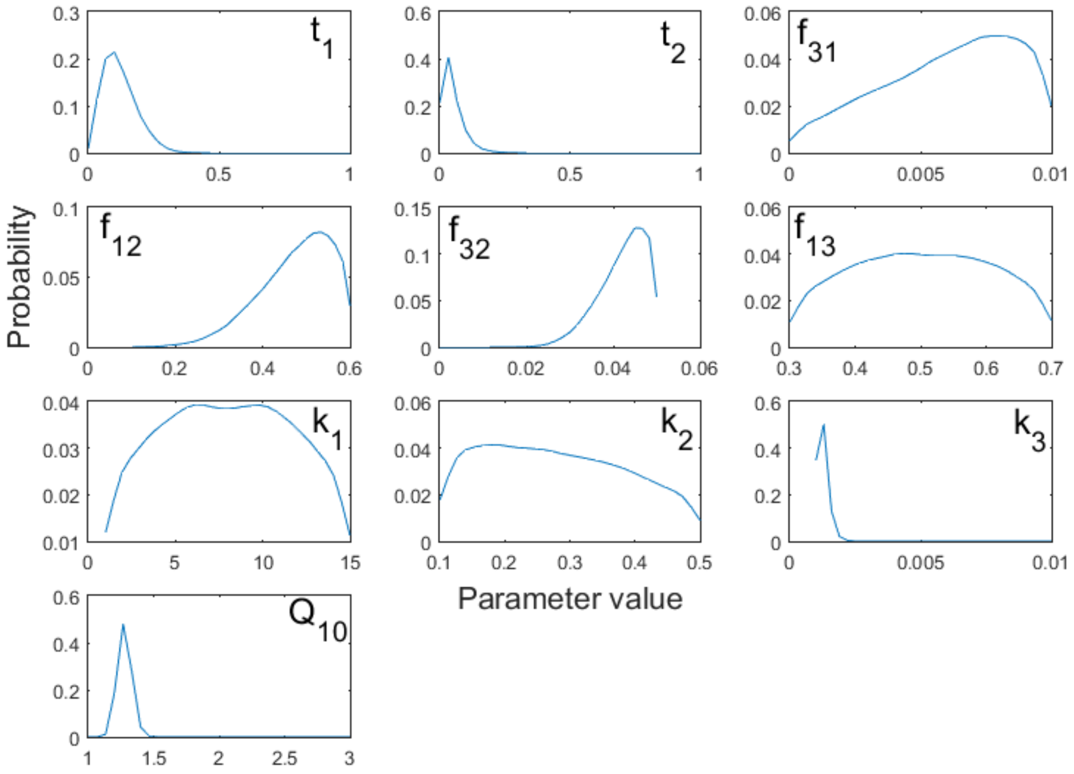

Supplementary Figure 4. The posterior probability distribution of the model parameters in the conventional model. Please refer to Table 1 for parameter meanings, range and default values.

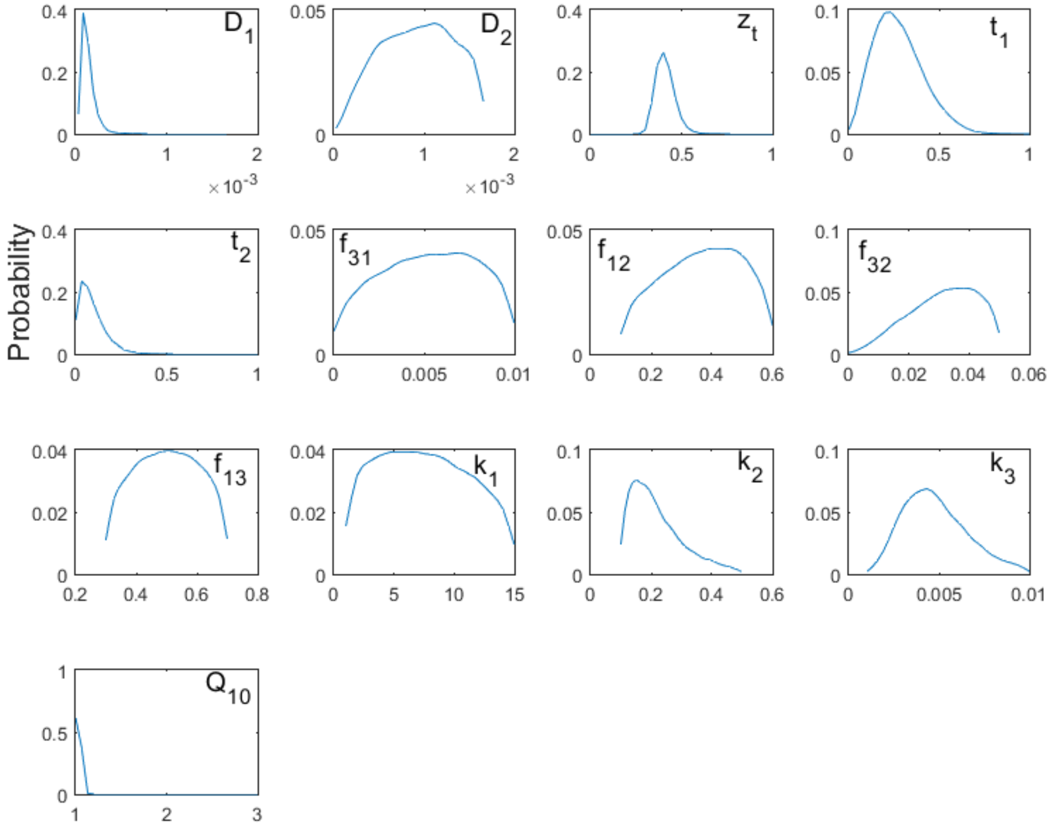

Supplementary Figure 5. **The posterior probability distribution of the model parameters in the vertically-resolved model.** Please refer to Table 2 for parameter meanings, range and default values in the CLM 4.5.

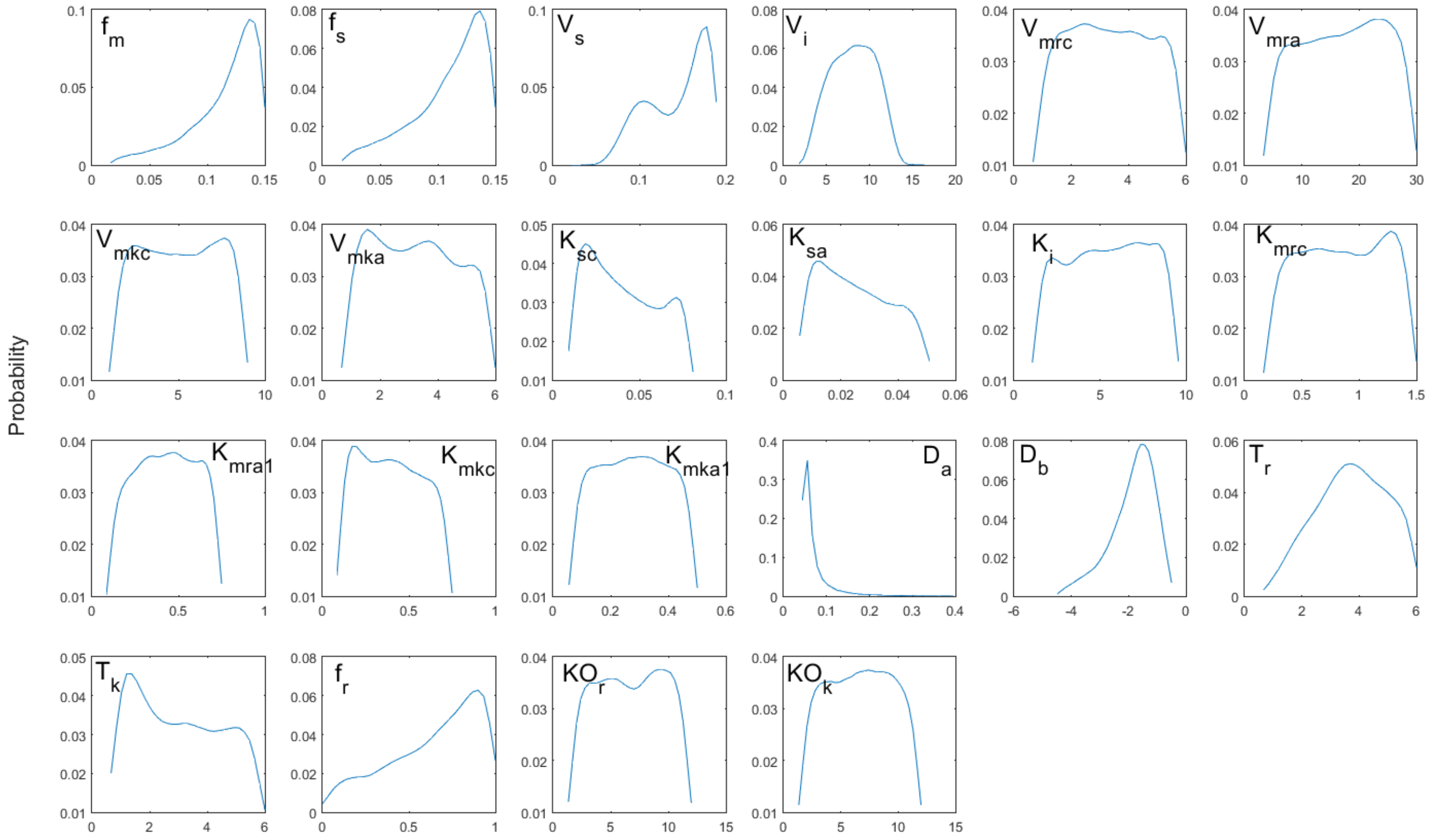

Supplementary Figure 6. The posterior probability distribution of the model parameters in the microbial model. Please refer to Table 3 for parameter meanings, range and default values in MIMICS.

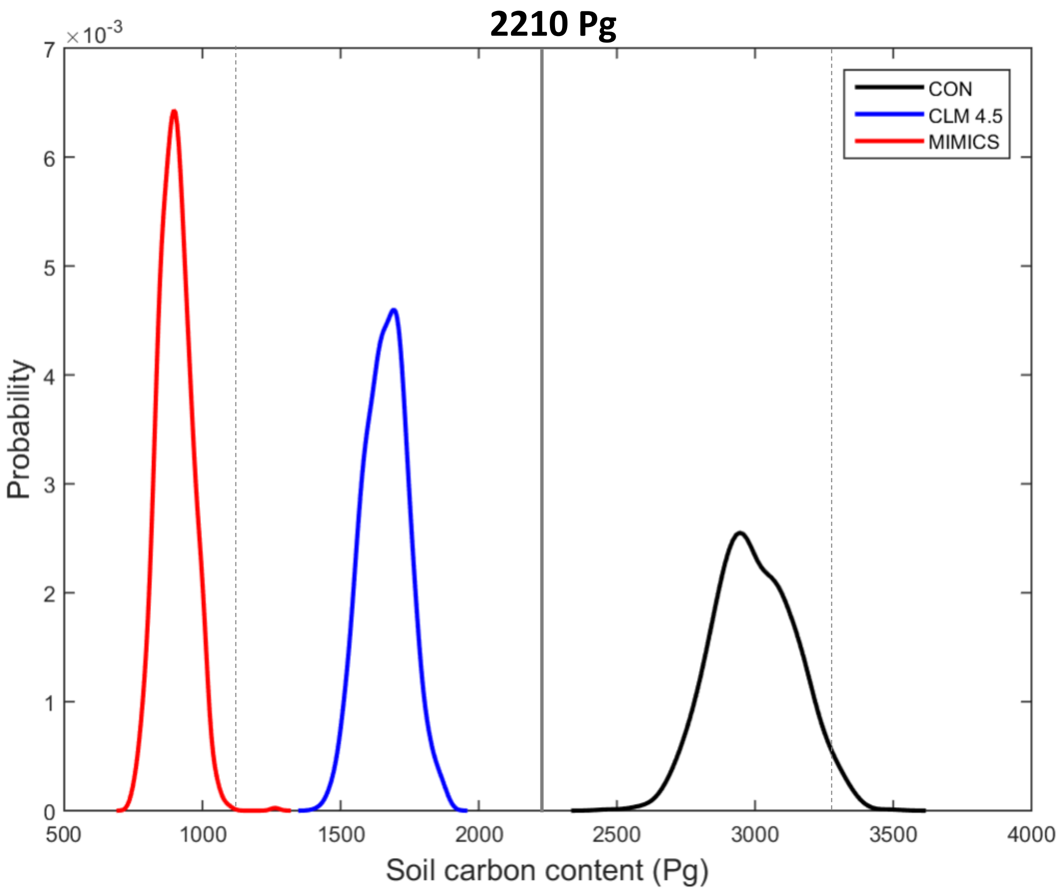

Supplementary Figure 7. Probability distribution of global total soil carbon at steady state in the three models. The estimated soil carbon was compared with the total soil carbon in the soil database (composite database with HWSD and NCSCD: 2210 Pg). CON in the legend is conventional Century-type model. Note that the total soil carbon in the vertically-resolved model is down to 1 meter in non-permafrost soils and down to 3 meters in the permafrost soils for the direct comparison with observation. Dashed lines are the range of one standard deviation of the observation.

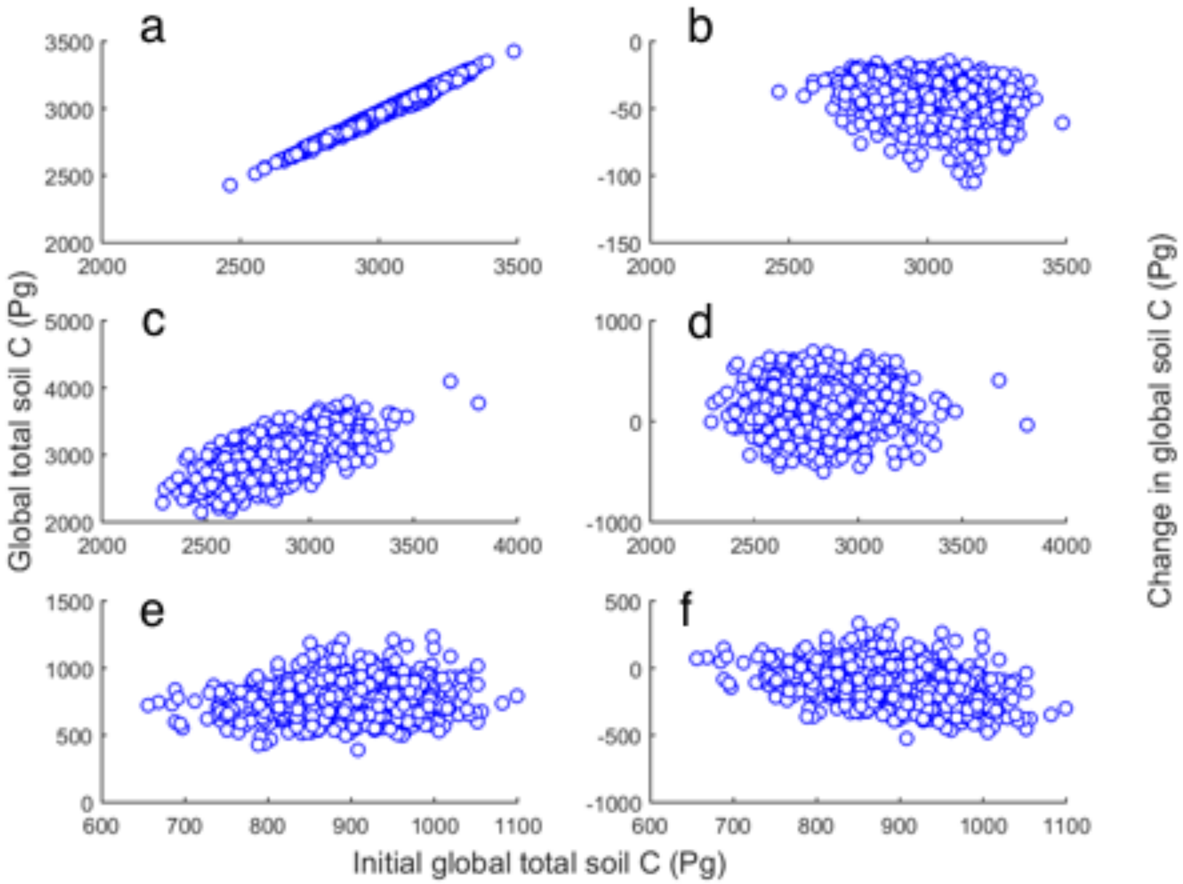

Supplementary Figure 8. Relationships between initial soil carbon condition and soil carbon prediction. Correlations between initial conditions of global total soil carbon and projected global total soil carbon in the last year and predicted changes in soil carbon under RCP 8.5 in the conventional model (a, b), vertically-resolved model (c, d) and the MIMICS (e, f). The correlation is calculated across the ensemble of 1,000 global simulations.

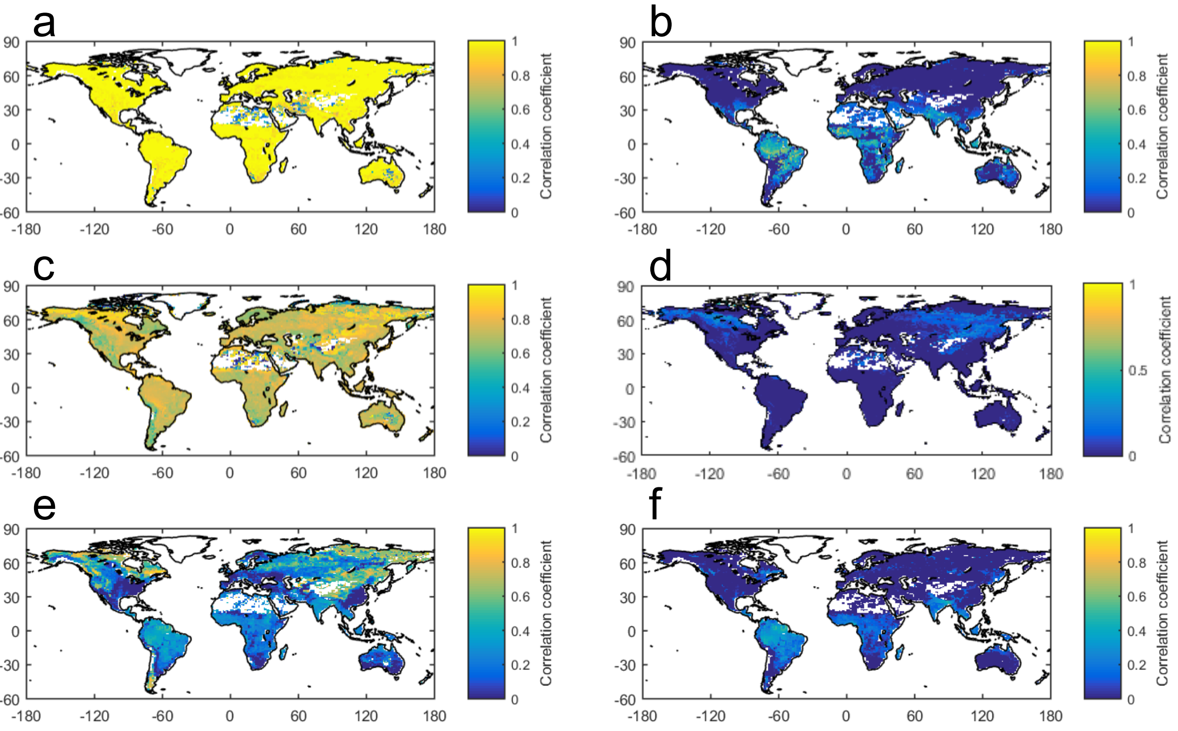

Supplementary Figure 9. Relationships between initial soil carbon condition and soil carbon prediction. Correlations between initial conditions of global total soil carbon and projected global total soil carbon in the last year and predicted changes in soil carbon under RCP 8.5 in each grid in the conventional model (a, b), vertically-resolved model (c, d) and MIMICS (e , f). The correlation is calculated across the ensemble of 1,000 global simulations.

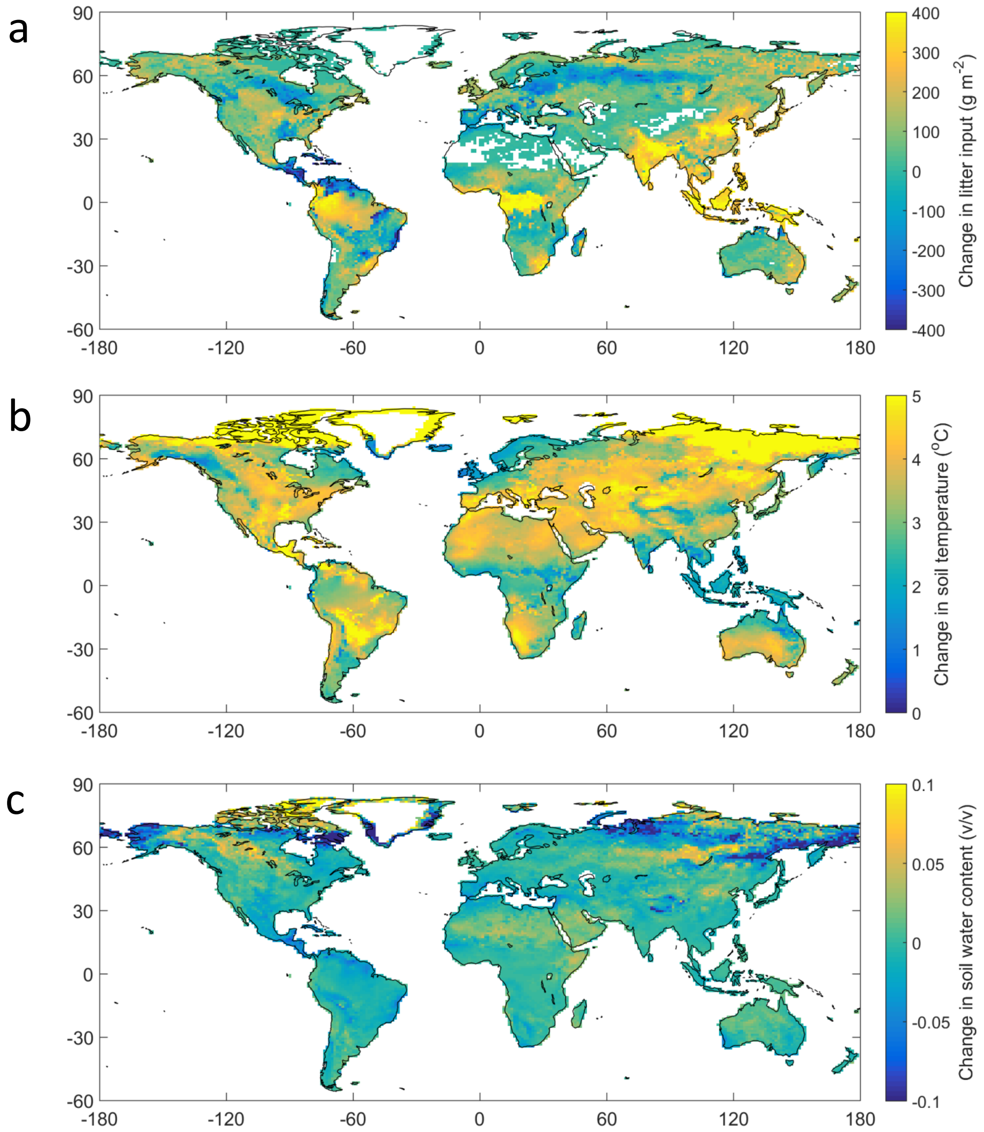

Supplementary Figure 10. Spatial changes in litter input (a), soil temperature (b) and soil water content (c) in CLM 4.5 within 2005-2100 under RCP 8.5.

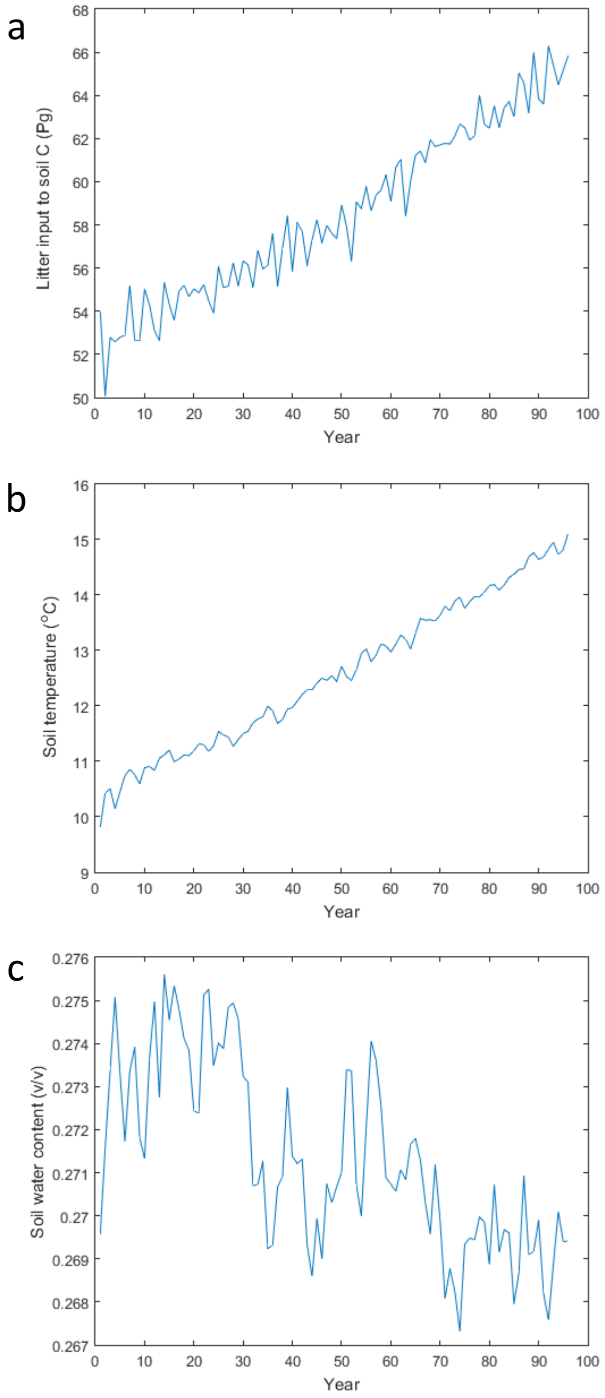

Supplementary Figure 11. Temporal changes in litter input (a), soil temperature (b) and soil water content (c) in CLM 4.5 within 2005-2100 under RCP 8.5.

Supplementary Table 1 Descriptions of parameters in the Microbial-Mineral Carbon Stabilization model. Parameter names, ranges, units, default values, posterior mean and standard deviation (SD), and G-R statistics in the microbial model, MIMICS. LL: lower limit; UL: upper limit

Parameter NO.	Description	Unit	LL	UL	Default value	Mean±SD	G-R
f_m	fraction of soil C input transferred to physically-protected SOM	-	0.02	0.15	0.05	0.11±0.029	1.01
f_s	fraction of soil C input transferred to chemically recalcitrant SOM	-	0.02	0.15	0.05	0.11±0.031	1.01
V_s	regression coefficient for calculating maximum reaction rate	ln(mg Cs(mg MIC)$^{-1}$ h^{-1})^{o}C^{-1}	0.02	0.189	0.063	0.14±0.035	1.01
V_i	regression intercept for calculating maximum reaction rate	ln(mg Cs(mg MIC)$^{-1}$ h^{-1})	1.82	16.41	5.47	8.09±2.56	1.00
V_{mrc}	modifies Vmax for fluxes into r-selection microbial biomass from chemically recalcitrant soil C to available soil C	-	0.67	6	2.00	3.35±1.46	1.00
V_{mra}	modifies Vmax for fluxes into r-selection microbial biomass from available soil C	-	3.33	30	10.00	16.97±7.37	1.00
V_{mkc}	modifies Vmax for fluxes into k-selection microbial biomass from structural litter or chemically recalcitrant soil C to available soil C	-	1.00	9	3.00	5.05±2.24	1.00
V_{mka}	modifies Vmax for fluxes into k-selection microbial biomass from available soil C	-	0.67	6	2.00	3.27±1.48	1.01

K_{sc}	regression coefficient for calculating half saturation constant for chemically recalcitrant soil C	ln(mg C cm^{-3}) $^{o}C^{-1}$	0.01	0.09	0.03	0.042±0.021	1.01
K_{sa}	regression coefficient for calculating half saturation constant for available soil C	ln(mg C cm^{-3}) $^{o}C^{-1}$	0.01	0.06	0.02	0.026±0.012	1.01
K_{i}	regression intercept for calculating half saturation constant	ln(mg C cm^{-3})	1.06	9.57	3.19	5.38±2.38	1.00
K_{mrc}	modifies Km for fluxes into r-selection microbial biomass from chemically recalcitrant soil C to available soil C	-	0.17	1.5	0.50	0.85±0.37	1.01
K_{mra1}	coefficient for modifying Km for fluxes into r-selection microbial biomass from available soil C	-	0.08	0.75	0.25	0.42±0.18	1.01
K_{mkc}	modifies Km for fluxes into k-selection microbial biomass from structural litter or chemically recalcitrant soil C to available soil C	-	0.08	0.75	0.25	0.40±0.18	1.01
K_{mka1}	coefficient for modifying Km for fluxes into k-selection microbial biomass from available soil C	-	0.06	0.51	0.17	0.28±0.12	1.00
D_{a}	coefficient a for calculating desorption rate from physically protected soil C to available soil C	h^{-1}	0.04	0.39	0.13	0.070±0.037	1.01
D_{b}	coefficient b for calculating desorption rate from physically protected soil C to available soil C	-	-0.50	-4.5	-1.50	-1.87±0.79	1.01

T_r	turnover rate of r-selection microbes	g C g C^{-1} year^{-1}	0.67	6	2.00	3.71±1.24	1.00
T_k	turnover rate of k-selection microbes	g C g C^{-1} year^{-1}	0.67	6	2.00	3.09±1.53	1.02
f_r	proportion of r-selection microbial biomass	-	0.17	1	0.50	0.64±0.26	1.00
KO_r	modifies Km for oxidation of chemically recalcitrant soil C for r-selection microbes	-	1.33	12	4.00	6.71±2.94	1.00
KO_k	modifies Km for oxidation of chemically recalcitrant soil C for k-selection microbes	-	1.33	12	4.00	6.68±2.90	1.01
